# Supplementary material for: Causal interplay between lactose intolerance and gut microbiota: a combined bidirectional Mendelian randomization and in vivo validation study
Source: Front Nutr. 2026 Jun 1;13:1803337. doi: 10.3389/fnut.2026.1803337 (PMC13265576; doi:10.3389/fnut.2026.1803337)
Supplement: Supplementary file 1 [file Data_Sheet_1.zip › supplementary materials/Forward/leave one out cross validation/ebi-a-GCST90027578.finngen_R12_E4_LACTONAS.pdf]

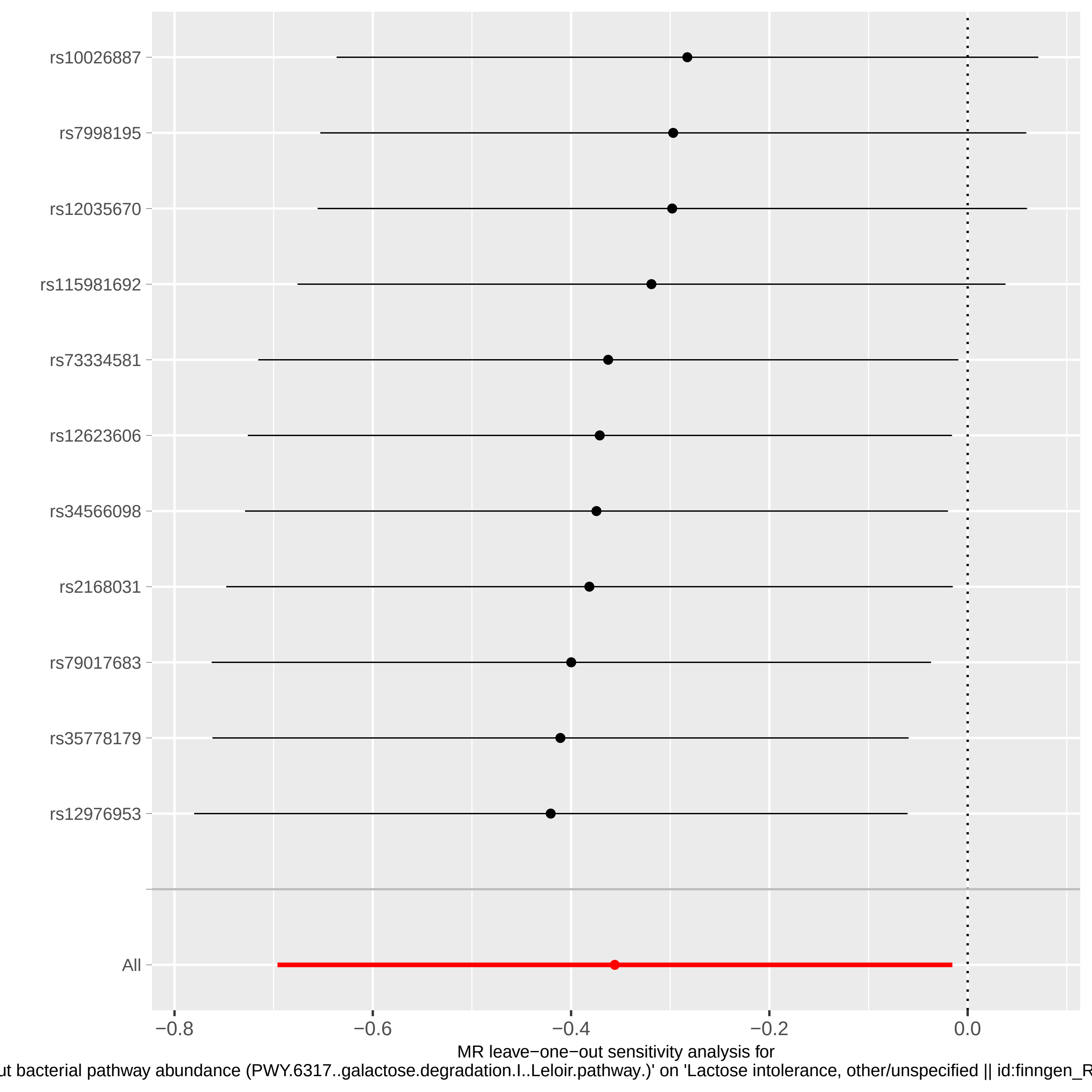

rs10026887
rs7998195
rs12035670
rs115981692
rs73334581
rs12623606
rs34566098
rs2168031
rs79017683
rs35778179
rs12976953
All
−0.8
−0.6
−0.4
−0.2
0.0
MR leave−one−out sensitivity analysis for
ut bacterial pathway abundance (PWY.6317..galactose.degradation.I..Leloir.pathway.)' on 'Lactose intolerance, other/unspecified || id:finngen_R
